# Transcriptomic Responses to Water Deficit and Nematode Infection in Mycorrhizal Tomato Roots

**DOI:** 10.3389/fmicb.2019.01807

**Published:** 2019-08-13

**Authors:** Raffaella Balestrini, Laura C. Rosso, Pasqua Veronico, Maria Teresa Melillo, Francesca De Luca, Elena Fanelli, Mariantonietta Colagiero, Alessandra Salvioli di Fossalunga, Aurelio Ciancio, Isabella Pentimone

**Affiliations:** ^1^Consiglio Nazionale delle Ricerche, Istituto per la Protezione Sostenibile delle Piante, Turin, Italy; ^2^Dipartimento di Scienze della Vita e Biologia dei Sistemi, Università di Torino, Turin, Italy

**Keywords:** abiotic stress, AM symbiosis, RKN, transcriptomics, stress response

## Abstract

Climate changes include the intensification of drought in many parts of the world, increasing its frequency, severity and duration. However, under natural conditions, environmental stresses do not occur alone, and, in addition, more stressed plants may become more susceptible to attacks by pests and pathogens. Studies on the impact of the arbuscular mycorrhizal (AM) symbiosis on tomato response to water deficit showed that several drought-responsive genes are differentially regulated in AM-colonized tomato plants (roots and leaves) during water deficit. To date, global changes in mycorrhizal tomato root transcripts under water stress conditions have not been yet investigated. Here, changes in root transcriptome in the presence of an AM fungus, with or without water stress (WS) application, have been evaluated in a commercial tomato cultivar already investigated for the water stress response during AM symbiosis. Since root-knot nematodes (RKNs, *Meloidogyne incognita*) are obligate endoparasites and cause severe yield losses in tomato, the impact of the AM fungal colonization on RKN infection at 7 days post-inoculation was also evaluated. Results offer new information about the response to AM symbiosis, highlighting a functional redundancy for several tomato gene families, as well as on the tomato and fungal genes involved in WS response during symbiosis, underlying the role of the AM fungus. Changes in the expression of tomato genes related to nematode infection during AM symbiosis highlight a role of AM colonization in triggering defense responses against RKN in tomato. Overall, new datasets on the tomato response to an abiotic and biotic stress during AM symbiosis have been obtained, providing useful data for further researches.

## Introduction

Drought is a devastating environmental condition that dramatically affects plant growth and crop production. Its adverse impact on global food security is mostly severe in semi-arid and arid regions from many parts of the world (Boyer, 1982). Additionally, climate changes are intensifying the frequency, duration and severity of drought in many agro-environments. In response to drought, domesticated plants rely on a number of physiological and structural adaptations to counteract water deficit or at least to escape most severe effects, as a result of selection for local cropping environments. The understanding of these adaptive mechanisms may be useful to sustain crop production, as well as for developing future breeding strategies. In addition to leaves, also roots, which are the first organ to detect a water deficit, are subjected to several modifications under drought, increasing water uptake and regulating water traffic between plant and soil (Gamboa-Tuz et al., 2018).

Water limiting conditions lead to changes in the expression of drought responsive genes in different plant tissues (Shinozaki and Yamaguchi-Shinozaki, 2007). Advances in technology, i.e., the development of -omics approaches, flanked by a parallel progress for *in silico* analyses, allowed the elucidation of the molecular mechanisms involved in the response to water deficit, in model and non-model plants. These include several economically important crops such as maize, rice, poplar, tomato, wheat or tropical fruits such as papaya (Kakumanu et al., 2012; Oono et al., 2014; Barghini et al., 2015; Gamboa-Tuz et al., 2018; Iovieno et al., 2018).

Tomato (*Solanum lycopersicum* L.) is one of the major horticultural crops cultivated worldwide and is a key component of the diet of many billion people. It has been reported that modern cultivars are sensitive to water deficit, which leads to a reduction in seed development and germination, impairing vegetative growth and reproduction (Iovieno et al., 2018). These authors exposed tomato plants to two cycles of prolonged drought stress and a single recovery by re-watering. Transcriptome datasets were generated for multiple time points during the stress and recovery cycles. Results allowed the identification and comprehension of the coordinated responses taking place under drought stress and subsequent recovery in leaves, highlighting the transcriptomic changes that control such physiological modifications (Iovieno et al., 2018).

Apart of abiotic stressors, plants interact in their environment with a complex soil and epiphytic microbiome, including both beneficial and noxious microorganisms. The mutualistic symbiosis established by arbuscular mycorrhizal (AM) fungi with the roots of most crops, have an important role in sustaining yields, as they act as bio-fertilizers and bio-protectors against both abiotic and biotic stresses (Balestrini et al., 2018). The latter include several soil pathogens and pests including plant parasitic nematodes (Schouteden et al., 2015). Root-knot nematodes (RKN; *Meloidogyne* species) are among the most devastating plant-parasitic nematodes (Jones et al., 2013). Having a wide host range, they cause large economic losses in cultivated plants that are expected to increase as a result of climate change leading water depletion and crop systems intensification. The increasing concern about the environmental impact of traditional nematicides have stimulated research for alternative control practices, including the use of biological control organisms. AM fungi have been reported to be effective against different pathogens and pests (Selosse et al., 2014; Martinez-Medina et al., 2016) and could represent a new environmental-friendly strategy to control nematode infection (Elsen et al., 2008; Vos et al., 2012; Schouteden et al., 2015; Sharma and Sharma, 2017).

Tomato has already been used as a crop model to study AM-colonized plants (Balestrini et al., 2018) as well as nematode–plant interactions (Portillo et al., 2013; Iberkleid et al., 2015; Shukla et al., 2018). Large-scale gene expression analyses have been carried out in mycorrhizal tomato plants using microarrays to identify genes differentially expressed (DE) in roots and shoots of AM-colonized plants (Fiorilli et al., 2009), as well as during the early interaction stages (Dermatsev et al., 2010). A deep sequencing of root transcriptome, using a wild-type tomato and a mutant incapable of supporting a functional AM symbiosis, showed the expression of several genes associated to AM symbiosis (Ruzicka et al., 2013). Comparison between transcriptomic profiles of tomato and *Lotus* AM-colonized roots has also been performed, suggesting that a certain proportion of AM-responsive genes are conserved across plant species (Sugimura and Saito, 2017). The authors also highlighted the fact that species-dependent AM-responsive genes might be related to specific root features, characterizing each host plant.

Several studies have been carried out on the impact of the AM symbiosis on the tomato response to water deficit (Dell’Amico et al., 2002; Subramanian et al., 2006; Aroca et al., 2008; Wang et al., 2014; Chitarra et al., 2016; Ruiz-Lozano et al., 2016; Rivero et al., 2018; Volpe et al., 2018). Targeted approaches already showed that several drought-responsive genes are differentially regulated in AM-colonized tomato plants (roots and leaves) during water deficit (Chitarra et al., 2016; Ruiz-Lozano et al., 2016). However, global changes in transcripts of mycorrhizal tomato roots, as affected by a water stress condition, have not yet been investigated, as this interaction was thus far studied only in AM-colonized bean roots (Recchia et al., 2018). We hence studied changes in the whole root transcriptome of *S. lycopersicum cv* San Marzano nano, which was previously tested to evaluate the impact of the AM symbiosis on the tomato water stress responses (Chitarra et al., 2016; Volpe et al., 2018). These previous works showed that the AM symbiosis positively affects the tomato tolerance to water deficit and how the adaptive plant response is dependent on the AM fungal species involved. Additionally, Volpe et al. (2018) have evaluated the impact of the colonization on tomato plants subjected to combined stresses (moderate water stress and aphid infestation) in controlled conditions. A positive effect on the tomato indirect defense toward aphids in terms of enhanced attractiveness toward their natural enemies was observed, as also supported by the characterization of volatile organic compound (VOC) released. In the present study, new information on the role of AM symbiosis to enhance crop tolerance to abiotic and biotic stresses in a global climatic change scenario has been obtained. In detail, changes induced in the transcriptome profile in roots colonized with the AM fungus *Rhizophagus intraradices* (i) in the presence of a moderate-water stress (abiotic stress), and (ii) following parasitism by the root-knot nematode *Meloidogyne incognita* (biotic stress) have been evaluated.

## Materials and Methods

### Plant Material and Growth Conditions

The *S. lycopersicum* ‘San Marzano nano’ genotype, important for its consumption in Italy, was used. Tomato seeds were surface sterilized in sodium hypochlorite for 20 min, washed five times in sterile water, and germinated on wet paper. Seedlings were then moved to pots containing a mixture of quartz sand (50%), sterile pumice (20%), and an inoculum (30%) of *R. intraradices* (FR 121), containing AM fungal propagules (spores, mycelium and mycorrhizal root pieces) in a carrier of mixed inert mineral, purchased from MycAgro Lab (Dijon, France). For non-inoculated plants, the sterile inoculum carrier was used alone, instead of the specific inoculum. The plants were maintained in a growth chamber under controlled conditions at 25°C, with a light intensity of 150 μmol/m^2^/s and a 16 h:8 h light/dark cycle.

#### Water Stress Treatment

Plants were abundantly irrigated with filtered tap water (twice a week) and Long Ashton solution containing 300 μM of inorganic phosphate (once a week) for 35 days prior the imposition of a moderate water stress treatment. Considered treatments were: (i) AM fungal colonization [non-colonized (C), *R. intraradices*-colonized (AM)] and (ii) water stress [none (unstressed or NS), moderate (WS) as described in Volpe et al. (2018)]. Nine replicates for each group (C, AM, WS, AM_WS) have been used, arranged in a randomized block design. Before starting the treatments, pots were weighed. Control plants were regularly watered throughout the entire experimental period whereas plants to be stressed were not watered until the pots reached a loss of about 210–220 g of the initial weight, a loss previously described to be needed to reach a moderate WS condition (Volpe et al., 2018). From this moment the plants received the amount of water or nutritive solution required to get them back to their last weight, in order to maintain a moderate stress level. At the end of the experiment (i.e., after 9 weeks), roots were sampled and 60 randomly chosen 1-cm-long root segments per 2 plants were stained with 0.1% cotton blue in lactic acid, to evaluate the presence of the AM fungus, before the RNA extraction. Due to the low quantity of root materials, mainly in the WS treatments, AM fungal colonization has been only qualitatively evaluated.

#### Nematode Infection Assay

Control and AM-colonized 8-week-old plants were inoculated with 1200 freshly hatched juveniles of *Meloidogyne incognita* per plant. Juveniles were collected from egg masses of infested tomato roots, which were allowed to hatch in water in a growth chamber at 25°C. At 7 days post-nematode inoculation (dpi) roots were harvested. Root galls from infected colonized (RKN_AM) and uncolonized (RKN) tomato plants were hand-picked and pooled in two biological replicates for each treatment. Samples were immediately frozen in liquid nitrogen and stored at −80°C until RNA-Seq experiments.

### Histological Observations

Galls at 7 dpi from AM-colonized and non-colonized tomato roots were hand-dissected under a stereomicroscope. At least five to ten galls were excised from each plant and fixed in a mixture of 1.5% glutaraldehyde and 3% paraformaldehyde (Sigma-Aldrich), dehydrated in ethanol and embedded in acrylic resin LR White (Sigma, St. Louis, MO, United States) according to Melillo et al. (2014). Embedded galls were cut in serial cross-semithin sections (2.5 μm) through all their length, then stained briefly with 1% toluidine blue in 1% borax solution and mounted in Depex. Microscopic observations were performed using bright-field optics on a Leica DM 4500 B light microscope equipped with a Leica DFC 450C camera.

### RNA Extraction and Sequencing

Six different treatments (C, WS, AM, RKN, AM_WS, RKN_AM) were set up to study the response in tomato roots. Total RNAs were extracted from root samples (two independent replicates for each treatment) using a RNeasy Plant Mini Kit (Qiagen, La Jolla, CA, United States) with addition of an on-column DNase I digestion, following the manufacturer’s protocol. RNA quantity and quality were determined with a Nanodrop 2000 spectrophotometer (Thermo Fisher Scientific Inc., Wilmington, DE, United States) and sent to IGA Technology Services (Udine, Italy)^1^. cDNA libraries were prepared from 4 μg total RNA using TruSeq RNA Sample Preparation Kit v2 (Illumina, Inc., San Diego, CA, United States) and validated according to Illumina’s low-throughput protocol. After normalization, cDNA libraries were pooled for multiplexing before loading onto a flow cell (8–9 samples per lane). The hybridization and cluster generation were performed on a cBot System using TruSeq SR Cluster Kit v3. Sequencing was performed on an Illumina HiScanSQ platform using TruSeq SBS kit v3 (Illumina, Inc.) to obtain Single Reads, 50 nt in length. Due to the scarcity of the collected material in WS treatment, and according to ENCODE standard for RNAseq experiments^2^, requiring at least two biological replicates, data analysis has then been performed only on C, AM, AM_WS, RKN and RKN_AM. Raw data have been deposited to NCBI and the accession number for this project is PRJNA545411.

### RNA-Seq Data Analysis and Differential Gene Expression Quantification

The quality of the raw sequence reads was checked using FastQC^3^. Raw sequences were processed to eliminate adapters, indexes, as well as genomic sequences added during the sequencing process, using the “RNA-seq analysis” functions included in the CLC Genomics Workbench software v.8.5 (QIAGEN, Aarhus, Denmark)^4^. Filtered reads from each sample were then separately aligned to the reference genome of *S. lycopersicum* (SL2.40.26, Sol Genomics Network)^5^, using CLC (similarity parameter: 0.8; identity parameter: 0.8, mismatch/insertion/deletion penalties: 2/3/3) and employed to quantify the abundance of tomato gene transcripts, measured as the Reads Per Kilobase per Million mapped reads (RPKM) (Mortazavi et al., 2008). A gene was considered to be expressed and included in the downstream analysis if at least five reads were mapped to it and its RPKM value was >0.

A multiple correlation test (Pearson’s correlation) on RPKM values for all pairwise combinations was performed for preliminary batch comparisons of replicates. To identify differentially expressed gene (DEG), statistical analysis was carried out for each treatment group against a reference group (equivalent developmental stages of un-colonized un-stressed roots). RNA-Seq library set differential expression analysis was performed applying “Empirical analysis of Digital Gene Expression (DEG) in R” (EDGE) that implements, in the EdgeR Bioconductor package (Robinson et al., 2010), the ‘Exact Test’ for two-group comparisons (Robinson and Smyth, 2008), able to account for over-dispersion caused by biological variability. Raw counts for each gene were normalized in relation to different sequence depths between duplicate bioassay samples.

Genes in libraries were considered DE when compared with the controls, by applying the Benjamini–Hochberg algorithm for Fold Change (FC) estimation. DEGs displaying at least a *P*-value (*p*) ≤ 0.05, in almost one condition, were submitted to further analysis. The terms up-regulation and down-regulation indicate, respectively, transcript levels that were significantly higher or lower than those observed in non-inoculated controls.

### Functional Analysis of Tomato DEG

Enrichment analysis of each DEG gene ontology (GO) term was performed by AgriGO device^6^ (Du et al., 2010) to identify GO category related to single or multiple interactions. GO Enrichment analysis detect functional categories of biological processes, molecular functions and cellular components over-represented, with statistical significance (Fisher’s Exact Test: *p*-value ≤ 0.05; Hochberg Multi-test adjustment method: FDR ≤ 0.05), in a gene sub set using annotations for that gene set as compared with the remaining genes of the reference organism (*S. lycopersicum* cDNA library, version 2.4^7^.

### Identification of AM Fungal Transcripts in Tomato Roots

To discover AM fungal genes expressed in roots, fungal reads were separated from those of tomato by mapping the complete RNA-Seq data set of AM-colonized roots onto the draft genome sequence of tomato version SL.2.46. Unmapped reads, potentially fungus-derived, were isolated and mapped, with CLC utility and parameter previously described, to the *Rhizophagus irregularis* DAOM 181602 v1.0 (formerly *Glomus intraradices*, GLOIN) genome, retrieved from JGI genome portal^8^ (Tisserant et al., 2013). The RPKM were determined and used to estimate the GLOIN transcripts abundance. To assess differential gene expression between AM_WS and AM samples, proportions-based *Z*-test statistical analysis (Kal et al., 1999) was applied, assigning the samples different weights depending on library size (total counts). A GO functional enrichment analysis was conducted using Fisher’s exact test with a weight algorithm in AgriGo. The GO annotations of *R. irregularis* genes were obtained from the Mycocosm JGI genome portal (Tisserant et al., 2013)^8^.

## Results

To gain a comprehensive understanding of the mechanisms activated in mycorrhizal tomato roots under abiotic and biotic stress conditions, plants were subjected to a moderate water stress and to a 7-days long RKN infection. Transcriptome root profiles were obtained from C, AM, AM_WS treatments, while the biotic stress impact was studied using the infection structure (gall) from non-mycorrhizal (RKN) and mycorrhizal (RKN_AM) plants.

### Sequencing Data and Transcriptome Mapping

Transcriptome sequencing generated a total of 187,7⋅10^6^ reads of 50 bp in length, for all samples. Good-quality reads (99%) were retained, which were mapped onto the reference genome with CLC. In total, 76–97% of good-quality reads were mapped onto the *S. lycopersicum* genome (SL2.40.26 assembly) across all samples (Table 1). Expressed tomato genes arose from 16347 (AM_WS2_7) up to 22959 (RKN1_7) out of 34647 predicted genes in the reference genome (Table 1), using a cutoff value of RPKM > 0 to declare a gene as expressed. A high Pearson’s correlation coefficient (*r*) was observed between RPKM values of each sample replicates sequenced set (average *r* = 0.92) (Table 2).

**TABLE 1:** Statistics of good-quality reads mapped onto the tomato reference genomes.

	**Cleaned**	**Aligned reads**	**SL expressed transcripts**
**SAMPLE**	**reads^∗^10^6^**	**on SL2.40 (%)**	**(≥5 aligned reads)**
C1_7	4.66	97.89	18417
C2_7	14.65	97.53	20065
RKN1_7	54.62	97.11	22959
RKN2_7	14.3	96.48	20917
AM1_7	15.49	78.56	20443
AM2_7	8.63	76.95	20064
RKN_AM1_7	15.55	93.98	21648
RKN_AM2_7	43.43	96.37	22957
AM_WS1_7	14.68	90.64	21460
AM_WS2_7	1.75	86.79	16347

**TABLE 2:** Correlation (Pearson’s correlation coefficient) between RPKM values of biological replicate of each sample pairwise combinations of sample set.

**SAMPLE**		**r**
C	1 *vs.* 2	0.87
RKN	1 *vs.* 2	0.90
AM	1 *vs.* 2	0.95
RKN_AM	1 *vs.* 2	0.96
AM_WS	1 *vs.* 2	0.93

### Whole Transcriptome Profiles and Differentially Expressed Genes

**FIGURE 1:**
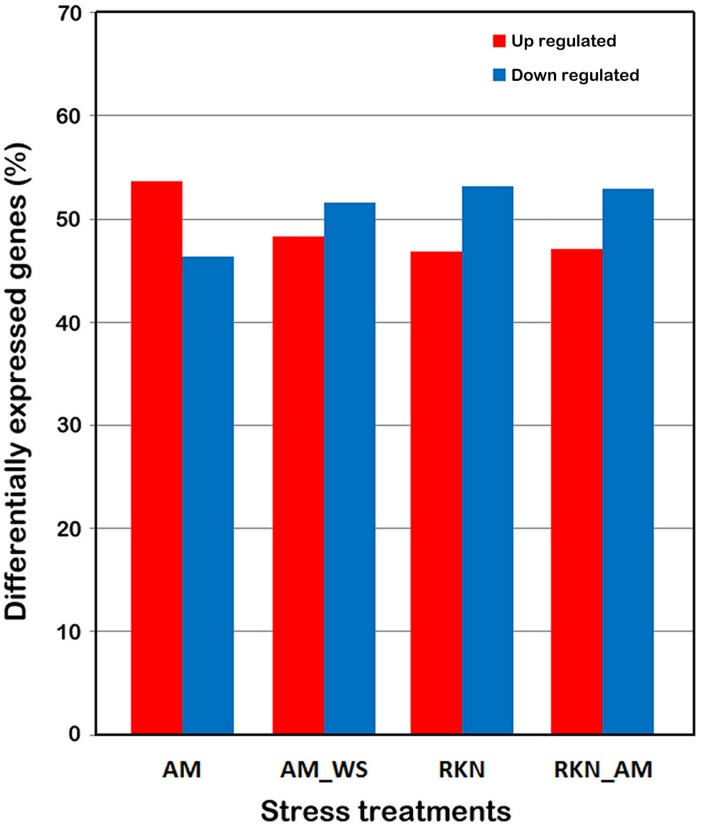
Differentially expressed tomato genes. DEGs were identified by comparing the expression profiles from treated roots (AM, AM_WS, RKN, RKN_AM) with the equivalent developmental stages of control roots (C) (*P* ≤ 0.05). Each column represents a treatment.

The genes DE respect to control (*p*-value < 0.05) arose from 10 (RKN) up to 12 % (AM) of total SL2.40.26 predicted protein coding genes (Table 3). A higher percentage of up-regulated genes was observed only in the AM condition (Figure 1). For further analyses, 7316 differentially expressed genes (DEGs), when compared with un-colonized/un-stressed control, and with a *p*-value < 0.05 in at least one treatment (AM, AM_WS, RKN, and RKN_AM) were considered (Supplementary Tables S1, S2).

**TABLE 3:** Differentially expressed tomato genes comparing to uninoculated unstressed plants (C) (*P* < 0.05).

**Condition**	**% DEG^∗^**	**Up regulated**	**Down regulated**
RKN	10	1659	1884
AM	12	2242	1938
RKN_AM	11	1855	2084
AM_WS	11	1905	2034

*^∗^Calculated using DEG out of 34647 predicted genes in the reference tomato genome.*

To have an overview of the regulation of the main metabolic processes and signaling pathways involved in the different stress conditions, a GO enrichment analysis was carried out for DEGs in AM_WS and RKN_AM. Data showed that transcripts involved in processes such as “response to oxidative stress,” “functions of peroxidase activity” and “heme binding” were involved in both situations. Specific GO terms enriched during abiotic stress (AM_WS) were related to the molecular function “transcription regulator activity,” while in biotic stress (RKN_AM) to the metabolic process “protein ubiquitination and protein amino acid phosphorylation” and cellular process “microtubule-based movement,” that were particularly over represented (Table 4, Supplementary Tables S3, S4, and Supplementary Figures S1, S2).

**TABLE 4:** GO Enrichment analysis (*P*-value ≤ 0.05) for differentially expressed genes during abiotic and biotic stress.

**Gene ontology**	**Categories**	**AM_WS**	**RKN_AM**
GO:0006979	P_response to oxidative stress	x	x
GO:0006950	P_response to stress	x	x
GO:0042221	P_response to chemical stimulus	x	x
GO:0050896	P_response to stimulus	x	x
GO:0016684	F_oxidoreductase activity, acting on	x	x
GO:0004601	F_peroxidase activity	x	x
GO:0020037	F_heme binding	x	x
GO:0005506	F_iron ion binding	x	x
GO:0046906	F_tetrapyrrole binding	x	x
GO:0016209	F_antioxidant activity	x	x
GO:0003824	F_catalytic activity	x	x
GO:0003700	F_transcription factor activity	x	
GO:0046914	F-transition metal ion binding	x	
GO:0005576	C_extracellular region	x	
GO:0004497	F_monooxygenase activity	x	
GO:0030528	F_transcription regulator activity	x	
GO:0046872	F_metal ion binding	x	
GO:0043167	F_ion binding	x	
GO:0055114	P_oxidation reduction	x	
GO:0016491	F_oxidoreductase activity	x	
GO:0043169	F_cation binding	x	
GO:0005524	F_ATP binding		x
GO:0032559	F_adenyl ribonucleotide binding		x
GO:0006464	P_protein modification process		x
GO:0006468	P_protein amino acid phosphorylation		x
GO:0006796	P_phosphate metabolic process		x
GO:0032555	F_purine ribonucleotide binding		x
GO:0016310	P_phosphorylation		x
GO:0004672	F_protein kinase activity		x
GO:0016772	F_transferase activity, transferring p-containing groups		x
GO:0043687	P_post-translational protein modification		x
GO:0007018	P_microtubule-based movement		x
GO:0016773	F_phosphotransferase activity, alcohol group as acceptor		x
GO:0005507	F_copper ion binding		x
GO:0016798	F_hydrolase activity, acting on glycosyl bonds		x
GO:0032553	F_ribonucleotide binding		x
GO:0016301	F_kinase activity		x
GO:0006793	P_phosphorus metabolic process		x
GO:0032446	P_protein modification by small protein conjugation		x
GO:0016567	P_protein ubiquitination		x
GO:0070647	P_protein modification by small protein conjugation or removal		x
GO:0043412	P_macromolecule modification		x

*Over-represented functional categories of biological processes (P), molecular functions (F) and cellular components (C).*

**FIGURE 2:**
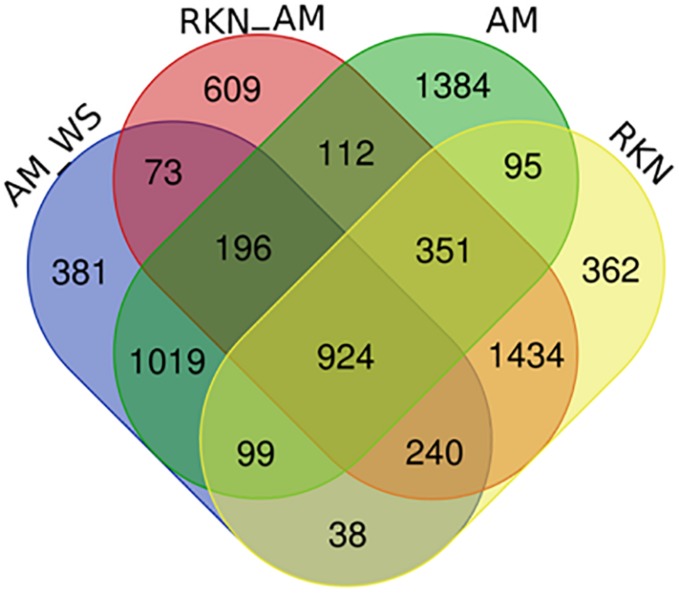
Venn diagram of DEGs (*P* ≤ 0.05) comparing un-inoculated un-stressed tomato (C) *vs.* AM, AM_WS, RKN, RKN_AM.

In the 7316 DEGs subset, a number of common and exclusive genes were observed among the treatments (Figure 2). Focusing on the 924 transcripts in common among AM, AM_WS, RKN, and RKN_AM treatments, 188 e 689 of them were up- and down-regulated, respectively, whereas the remaining 47 transcripts showed an opposite expression trend, in at least one condition (Supplementary Table S5). GO enrichment analysis conducted for this subset showed up regulated transcripts belonging to processes involved in “biotic stimulus,” “oxidative stress response” and functions like “heme binding,” “transition metal ion binding” and “peroxidase activity.” Processes such as “post-translational protein modification” and “regulation of transcription” were down-regulated, while transcripts ascribed to the “heme binding” function showed different expression trends (Supplementary Table S6). Looking at the AM fungal presence, 196 genes were DE in all the AM-colonized plants, independently from the applied stress condition (Figure 2).

Among the twenty most up-regulated genes found in each condition (Supplementary Table S7), all genes of AM_WS sample were also retrieved in the top 20 DEGs of unstressed AM-colonized plants (AM), although a lower fold change was often recorded in AM_WS plants, probably reflecting a low level of colonization in stressed plants with respect to the AM ones. One transcript (Solyc01g067860.2.1) was up regulated in all stress conditions. Considering the biotic stresses (RKN and RKN_AM), 12 transcripts were in common. The most up-regulated genes in the infection structures (galls) were mainly related to biotic stress response, independently from the AM fungal presence.

### Specific Responses to the AM Fungus in Drought Stressed *Versus* Unstressed Roots

To deeper explore this novel dataset and to further understand the tomato response to WS during AM interaction, as well as the impact of the imposed stress on the symbiosis, the expression profiles of genes described in the literature as specifically involved during AM symbiosis in different plant species have been considered (Fiorilli et al., 2009; Guether et al., 2009; Hogekamp et al., 2011; Hogekamp and Küster, 2013; Fiorilli et al., 2015, 2018; Handa et al., 2015; Balestrini et al., 2017; Li et al., 2018; Recchia et al., 2018; Vangelisti et al., 2018). Particularly, AM symbiosis is known for the improved nutrient exchange established between the two symbionts, involving fine-tuned plant and fungal transporter genes (Casieri et al., 2013; Berruti et al., 2016). A consistent group of plant transporters were identified as DE between unstressed colonized plants (AM) and the control (C) ones (Supplementary Table S8) also confirming that a functional symbiosis was established. Several transporters were significantly up-regulated in AM-colonized plants subjected to a moderate water stress (Supplementary Table S8 and Table 5). Two of them code for two inorganic phosphate transporters (Solyc09g090080.1.1 and Solyc06g051860.1.1), which show homology with the mycorrhizal inducible *LePT3* and *LePT4* (Balestrini et al., 2007). The other mycorrhiza-inducible *LePT5* (Solyc06g051850.1.1) was still up-regulated in AM_WS, although not in a significant way (*P* > 0.05), in agreement with the fact that this one was the AM-inducible PT gene significantly affected only by the “stress” factor (Volpe et al., 2018). Many other genes coding proteins involved in molecule transport were significantly up-regulated in the presence of the AM fungus upon water stress, such as three ammonium transporters (AMT; i.e., Solyc08g067080.1.1, Solyc03g033300.1.1, Solyc09g065740.1.1) and four potassium (K^+^) transporters (Solyc03g097860.1.1, Solyc06g051860.1.1 Solyc07g014680.2.1, Solyc05g005740.2.1). Tomato genes coding for three ABC transporter family G (Solyc11g007300.1.1, Solyc01g097430.2.1, Solyc09g098410.1.1), with the last two homologs of the AM symbiosis-induced ABCG transporter *LjSTR2* and *LjSTR*, were among the ten most up-regulated genes in AM_WS, in addition to the three AMT genes, two genes coding for peptide transporters (Solyc01g102360.1.1, Solyc05g009500.2.1) and one gene encoding an high affinity cationic amino acid transporter (CAT; Solyc12g096380.1.1). Sulfate transporters were also regulated and two of them were significantly induced by AM symbiosis (Solyc12g056920.1.1, Solyc10g047170.1.1). In AM_WS they were up- and down-regulated respectively, although not significantly different. Among the down-regulated genes in AM symbiotic roots, both in unstressed and stressed plants, there was a PT gene (Solyc09g066410.1.1) that shows homology with an inorganic phosphate transporter 1-4 (XP_004247235.1) expressed in tomato fruit from mycorrhizal plants (Zouari et al., 2014).

**TABLE 5:** Transporter genes significantly regulated by the AM fungus in non-stressed (NS) and stressed (WS) growth conditions.

**Transporter category**	**# of genes**		**Fold change mean**	
	**C *vs.***	**C *vs.***	**C *vs.***	**C *vs.***
	**AM**	**AM_WS**	**AM**	**AM_WS**
*ABC transporter G*	8	7	238.26	56.78
*Amino acid permease*	8	7	1.71	0.76
*Cationic amino acid transporter*	3	1	117.39	94.67
*Peptide transporters*	13	8	141.77	73.43
*Aquaporins*	15	11	10.80	4.62
*Putative SWEET transporter*	1	0	21.37	0.00
*Phosphate transporters*	4	3	85.76	30.31
*Potassium transporters*	7	4	22.88	10.42
*Ammonium transporters*	6	5	426.36	121.11
*Nitrate transporters*	6	4	–2.93	–12.66
*Sulfate transporters*	3	0	2.05	0.00
*Zinc transporters*	5	3	8.41	1.80
*Copper transporters*	1	7	75.80	20.07
*Glucose transporters*	8	7	–14.51	–9.74
*Lipid A export permease*	8	5	31.18	10.40

Almost all the genes reported by Sugimura and Saito (2017) as symbiosis marker genes both in tomato and *Lotus* were also found strongly up-regulated in AM-colonized roots (Supplementary Table S9), both in unstressed and AM_WS stressed plants. Additionally, other members within gene families already known to be involved in AM symbiosis (e.g., blue copper proteins, germin-like proteins, glutathione-*S*-transferase, ripening-related proteins, cell wall-related genes etc.) were strongly up-regulated in the presence of the AM fungus (Supplementary Table S10). Generally, expression levels were almost always lower in AM_WS than in AM roots, probably due to an opposite impact of the water stress on their regulation. Among the genes strongly up-regulated in AM_WS (i.e., with fold changes >50), there were those encoding three putative glutathione-*S*-transferases (Solyc02g081240.1.1, Solyc10g007620.1.1, Solyc10g084960.1.1) in addition to a putative germin-like (Solyc11g068570.1.1), three blue copper (Solyc12g056500.1.1, Solyc10g081520.1.1, Solyc08g079780.1.1), two major allergen (Solyc03g117460.1.1, Solyc03g117450.1.1), four ripening-related (Solyc01g094440.1.1, Solyc05g048780.1.1, Solyc01g094450.1.1, Solyc01g094430.1.1), two putative dirigent-like proteins (Solyc07g052170.1.1, Solyc04g010270.1.1) with three putative cysteine proteinases (Solyc12g056000.1.1, Solyc08g065710.1.1, Solyc08g065690.1.1), and two subtilisin-like proteases (Solyc08g080010.1.1, Solyc02g072370.1.1) (Supplementary Tables S9, S10).

Hormonal related transcript levels have been also already reported to change in the presence of the AM fungus (Gutjahr, 2014; Bedini et al., 2018; McGuiness et al., 2019). The AM marker gene *CCD7* (Solyc01g090660.2.1) coding for a carotenoid cleavage dioxygenase 7 with a role in strigolactone (SL) biosynthesis, was found to be still regulated in AM-colonized WS roots (AM_WS) in addition to *CCD8* gene (Supplementary Tables S5, S6). Gibberellin-related genes have been also found to be affected by AM symbiosis in *Medicago truncatula*. In agreement with the data in Ortu et al. (2012), tomato DELLA GAI protein (Solyc11g011260.1.1; Nir et al., 2017) resulted to be up-regulated in the AM treatment, while Gibberellin receptor GID1L2 (Solyc09g075670.1.1) was down-regulated. Gibberellin 20-oxidase-like proteins were up-regulated in AM-colonized roots (Solyc12g013780.1.1 and Solyc01g093980.2.1), while a putative Gibberellin 20-oxidase-1 (Solyc03g006880.2.1) was induced only in the AM treatment. Among the Gibberellin 2-oxidases, one gene (Solyc02g070430.2.1) resulted to be down-regulated in both AM-colonized roots, while Solyc07g061730.2.1 was significantly repressed only in AM_WS and Solyc07g061720.2.1 was up-regulated in AM roots (Supplementary Table S10). Interestingly, genes coding for enzymes with a key regulatory role in the biosynthesis of phenylpropanoid products, which can have diverse functions and are particularly important in plant defense (Mandal et al., 2010), were upregulated in AM plants. In detail, genes coding for two phenylalanine ammonia lyases (PAL; Solyc03g042560.1.1, Solyc09g007900.2.1) and a 4-coumarate-CoA ligase (4CL; Solyc11g007970.1) were significantly up-regulated in AM-colonized plants, suggesting an effect of the AM fungus for enhanced defense ahead of stress occurrence.

In agreement with data already reported (Liu F. et al., 2015), several non-specific lipid transfer proteins were also detected as significantly regulated during AM symbiosis also under WS condition (Supplementary Table S10). A core of differentially regulated genes was related to genes involved in cell wall metabolism and modification, in agreement with previous works (Balestrini and Bonfante, 2014). Among the genes putatively involved in arbuscule development and fungal accommodation, four genes coding for cellulose synthases were significantly up-regulated (Solyc00g030000.1.1, Solyc00g154480.1.1, Solyc07g051820.2.1, Solyc08g076320.2.1), independently from the stress level, in addition to an endo-β-1,4-glucanase (Solyc04g064900.1.1). Several genes encoding expansin proteins were also differentially up- or down regulated in both AM and AM_WS roots, confirming a role for these proteins during the AM colonization.

Transcription factors (TFs) have been reported to be highly involved in the response to drought as well as in AM symbiosis establishment. In our dataset, AM colonization, independently from the growth conditions, elicited the expression of several TF genes belonging to different groups, while other members inside these families were down-regulated (Supplementary Table S9). Looking at the genes expressed in AM and AM_WS, two ethylene responsive transcription factor 2a (Solyc04g071770.2.1, Solyc12g056590.1.1) resulted to be down-regulated, while an opposite trend was observed for the *LeERF5* (Solyc03g093560.1.1), up-regulated in AM-colonized roots, independently from the imposed stress. Due to the role in the adventitious roots and in the regulation of auxin responsive genes, the up-regulation in both AM and AM_WS roots of auxin responsive factor (ARF) genes (Solyc07g043610.2.1, Solyc03g118290.2.1) is worth noting since they are considered to be related to the modification of the root apparatus occurring during symbiosis (Vangelisti et al., 2018).

The expression of WS-responsive genes has been considered, to verify the efficacy of the WS stress imposition on tomato transcriptome in the presence of the AM fungus. The relevance of ABA in the response of plants to a water deficit event has already been highlighted by the differential regulation of target genes, directly associated with the ABA biosynthesis and signaling (Kuromori et al., 2018). The co-regulation with negative regulators has already been reported, suggesting the presence of a mechanism to fine-tune plants stress responses (Garcia et al., 2008). The presence of the fungus, independently from the growth condition (NS and WS), induced in roots the expression of a gene (Solyc07g056570.1.1, i.e., LeNCED1) coding a 9-*cis*-epoxycaratenoid dioxygenase involved in the ABA biosynthetic process, with a fold change value higher in AM than in AM_WS plants. A gene coding for a NAC transcription factor JA2 (Solyc12g013620), reported to be induced by ABA and WS in tomato leaves, and promoting stomatal closure through the induction of the expression of ABA biosynthetic gene NCED1, was down-regulated in AM-colonized roots, both in stressed and unstressed condition, suggesting a specific role in leaves. Solyc03g116390.2.1, coding for a late embryogenesis abundant (LEA) protein already shown to be inducible by drought stress (Gong et al., 2010) was significantly up-regulated in AM_WS, additionally to other two LEA genes (Solyc12g098900.1.1, Solyc10g078770.1.1) and the ABA-responsive gene coding for dehydrin TAS14 (Solyc02g084850.2.1; Sacco et al., 2013), confirming the efficacy of the imposed water stress. Additionally, Solyc06g019170.2.1, the ABA responsive gene Delta l-pyrroline-5-carboxylate synthetase SlP5CS1, involved in proline production, was significantly up-regulated only in AM_WS (Supplementary Tables S1, S2).

As expected, the several tomato aquaporin (AQP) genes showed different regulations among the considered conditions. Several AQP genes (Table 6) were significantly up- or down-regulate in AM roots, and many were significantly regulated also in AM_WS ones. The significant up-regulation observed for the AQP Solyc09g007770.2.1 (*SlPIP2;1*) in AM_WS roots is worth of noting, suggesting a specific role in WS response for this transcript.

**TABLE 6:** List of AQPs regulated in the several conditions (AM, unstressed AM-colonized roots; AM_WS, water stressed AM-colonized roots; RKN, nematode infection; RKN_AM, nematode infection in AM-colonized plants).

**Transcript ID**	**Gene name**	**AM**	**AM_WS**	**RKN**	**RKN_AM**
Solyc12g057050.1.1	*SlNIP3;2*	**121.30**	**36.08**	1.09	3.39
Solyc03g005980.2.1	*SlNIP1;1*	**12.26**	**4.90**	1.41	1.31
Solyc10g084120.1.1	*SlPIP2;5*	**9.32**	**4.76**	–1.01	1.63
Solyc06g011350.2.1	*SlPIP2;4*	**8.97**	**7.34**	–1.05	–1.17
Solyc06g060760.2.1	*SlTIP2;3*	**4.43**	1.90	**−4.59**	**−3.23**
Solyc08g081190.2.1	*SlPIP1;5*	**4.20**	**2.61**	1.59	1.79
Solyc12g056220.1.1	*SlPIP1;3*	**4.12**	2.00	1.46	1.63
Solyc12g019690.1.1	*SlSIP1;1*	**3.64**	**2.46**	1.35	1.39
Solyc11g069430.1.1	*SlPIP2;6*	**3.38**	**2.43**	1.73	1.82
Solyc10g083880.1.1	*SlTIP1;3*	**3.17**	**2.57**	**−2.98**	–1.38
Solyc03g013340.2.1	*SlNIP2;1*	**2.81**	–1.49	–1.52	1.15
Solyc06g074820.2.1	*SlTIP1;1*	2.62	**2.84**	1.14	1.50
Solyc06g075650.2.1	*SlTIP1;2*	1.94	1.82	**−3.00**	**−2.17**
Solyc08g008050.2.1	*SlPIP1;1*	1.79	1.26	**−5.83**	–2.68
Solyc09g007770.2.1	*SlPIP2;1*	1.33	**8.68**	–1.44	1.84
Solyc02g071920.2.1	*SlNIP1;2*	1.16	–1.71	**−3.82**	**−2.37**
Solyc01g094690.2.1	*SlPIP1;2*	1.01	1.80	**6.67**	**5.93**
Solyc06g073590.2.1	*SlNIP3;1*	–2.02	**−5.81**	**−11.36**	**−8.89**
Solyc10g078490.1.1	*SlSIP1;2*	**−2.21**	**−2.25**	–1.73	–1.48
Solyc08g066840.2.1	*SlTIP4;1*	**−2.38**	–1.65	–1.22	–1.21
Solyc12g044330.1.1	*SlTIP2;1*	**−5.43**	**−4.87**	–2.95	–2.06
Solyc03g096290.2.1	*SlPIP1;7*	**−5.59**	**−5.23**	1.68	1.29

*Values in bold are significantly regulated (*P* < 0.05).*

### AM Fungal Gene Regulation Upon Water Stress

Gene expression analysis of *R. irregularis* colonizing tomato roots was performed by mapping short reads against the *R. irregularis* genome. The proportion of *R. irregularis*-derived reads was less than 12% in AM roots and of 5% in AM_WS (Table 7), consistent with the results of previous studies (Tisserant et al., 2013; Handa et al., 2015; Sugimura and Saito, 2017). We identified 19057 and 16364 *R. irregularis* expressed genes (RPKM > 0), respectively, in AM and AM_WS roots, with 78.8% of overlapping, corresponding to 52% of the *R. irregularis* putative protein-coding genes expressed in both conditions. The expression levels of *R. irregularis* genes were significantly correlated between AM and AM_WS samples (Pearson correlation = 0.85). 77% of the *R. irregularis* genes found in tomato roots (RPKM > 0) coincided with those expressed by the fungus in *Lotus japonicus* roots, according to Handa et al. (2015). A total of 1224 *R. irregularis* genes, 4% of the entire transcriptome, were DE between AM and AM_WS treatments (FC ≥ 2; *p*-value ≤ 0,01), of which 779 were up-regulated and 445 down- regulated in roots subjected to moderate water stress (Supplementary Table S11). A GO enrichment analysis of the subset of genes highly expressed upon water stress revealed an overrepresentation of GO terms related to molecular function “monooxigenase activity” and “heme binding” (Supplementary Table S12). The most remarkable difference was the higher expression of 18 cytochrome P450 (CYPs), belonging to “heme binding” molecular function, 2 to 12-fold overexpressed in stressed roots (Supplementary Table S11). By contrast, only 5 CYP genes turned out to be down-regulated, with a mean fold change of −2.7 (Supplementary Table S11). Concerning the 20 most up- and down regulated genes, it is worth noting that the majority of them lacks a KOG annotation (16 out of 20 genes both for the up- and the down-regulated). However, the most up-regulated sequence (FC 74.5) contains a CsbD domain, that is present in a bacterial gene induced upon abiotic stresses such as nutrient-limitation (Prágai and Harwood, 2002). Another highly up-regulated gene (FC 65.23) contains a “conidiation protein 6” domain.

**TABLE 7:** Summary statistics for Illumina sequencing and mapping against reference *R. irregularis* DAOM198197 genome assembly (Gloin1).

	**AM**	**AM_WS**	**RKN_AM**
Total raw reads	24,243,262	16,586,731	59,231,940
Reads mapped against *R. irregularis* reference sequence	2,884,324	885,917	193,410
Mapped reads /total raw reads (%)	11.9	5.34	0.33

Several genes encoding proteins containing BTB/POZ and Kelch domains, involved in signaling transduction and regulation at the protein level, were also highly regulated in our dataset (mean up and down FC: 10.7 and −4.3, respectively). Genes encoding for proteins involved in protein turnover (ubiquitin pathway and chaperones) were DE, with 40 transcripts found as up-regulated. Remarkably, three sequences ascribed to glutathione S-transferase were up-regulated in the AM_WS treatment, none of them being down-regulated. As for *R. irregularis* genes down regulated in stressed tomato roots, they were fewer compared with the up-regulated ones, and no overrepresented GO terms was recorded (Table 8).

**TABLE 8:** Most relevant fungal DEGs.

**Fold change (mean value)**		**n°**	**Feature ID**
**Cytochrome P450**			
Up	4.04	18	jgi| Gloin1| 43670|;jgi| Gloin1| 322668|; jgi| Gloin1| 54382|; jgi| Gloin1| 36234|; jgi| Gloin1| 82149|; jgi| Gloin1| 35874|; jgi| Gloin1| 59731|; jgi| Gloin1| 57850|; jgi| Gloin1| 54172|; jgi| Gloin1| 348593|; jgi| Gloin1| 330761|; jgi| Gloin1| 70184|; jgi| Gloin1| 347608|; jgi| Gloin1| 74648|; jgi| Gloin1| 54229|; jgi| Gloin1| 337700|; jgi| Gloin1| 348383|; jgi| Gloin1| 1959|
Down	–2.75	5	jgi| Gloin1| 130232|; jgi| Gloin1| 21462|; jgi| Gloin1| 300912|; jgi| Gloin1| 51235|; jgi| Gloin1| 62820|
**Defense-related genes**			
Up	4.99	6	jgi| Gloin1| 350397|; jgi| Gloin1| 250811|; jgi| Gloin1| 20587|; jgi| Gloin1| 78868|; jgi| Gloin1| 23533|; jgi| Gloin1| 7020|
Down	–2.43	2	jgi| Gloin1| 18774|; jgi| Gloin1| 342859|
**Stress-related genes**			
Up	4.36	2	jgi| Gloin1| 342251|; jgi| Gloin1| 336918|
Down	–2.82	1	jgi| Gloin1| 342324|
**Vesicular trafficking**			
Up	2.97	9	jgi| Gloin1| 5531|; jgi| Gloin1| 347798|; jgi| Gloin1| 336277|; jgi| Gloin1| 28807|; jgi| Gloin1| 341557|; jgi| Gloin1| 348182|; jgi| Gloin1| 265701|; jgi| Gloin1| 289764|; jgi| Gloin1| 342517|
Down	–2.96	4	jgi| Gloin1| 174850|; jgi| Gloin1| 23226|; jgi| Gloin1| 30939|; jgi| Gloin1| 5044|
**Chaperones and protein turnover**			
Up	5.84	40	jgi| Gloin1| 113616|; jgi| Gloin1| 336528|; jgi| Gloin1| 46676|; jgi| Gloin1| 71363|; jgi| Gloin1| 16998|; jgi| Gloin1| 22115|; jgi| Gloin1| 340280|; jgi| Gloin1| 323494|; jgi| Gloin1| 46515|; jgi| Gloin1| 6786|; jgi| Gloin1| 345683|; jgi| Gloin1| 302299|; jgi| Gloin1| 5369|; jgi| Gloin1| 79494|; jgi| Gloin1| 16101|; jgi| Gloin1| 58870|; jgi| Gloin1| 52235|; jgi| Gloin1| 44858|; jgi| Gloin1| 57249|; jgi| Gloin1| 327|; jgi| Gloin1| 27674|; jgi| Gloin1| 66066|; jgi| Gloin1| 21968|; jgi| Gloin1| 349345|; jgi| Gloin1| 34571|; jgi| Gloin1| 299589|; jgi| Gloin1| 328597|; jgi| Gloin1| 53501|; jgi| Gloin1| 163681|; jgi| Gloin1| 345252|; jgi| Gloin1| 55552|; jgi| Gloin1| 66159|; jgi| Gloin1| 339540|; jgi| Gloin1| 24173|; jgi| Gloin1| 340241|; jgi| Gloin1| 345467|; jgi| Gloin1| 345863|; jgi| Gloin1| 343006|; jgi| Gloin1| 84344|; jgi| Gloin1| 311043|; jgi| Gloin1| 289764|
Down	–3.26	15	jgi| Gloin1| 106703|; jgi| Gloin1| 177771|; jgi| Gloin1| 188968|; jgi| Gloin1| 219634|; jgi| Gloin1| 330117|; jgi| Gloin1| 336698|; jgi| Gloin1| 338665|; jgi| Gloin1| 339110|; jgi| Gloin1| 345483|; jgi| Gloin1| 345595|; jgi| Gloin1| 348922|; jgi| Gloin1| 6665|; jgi| Gloin1| 69178|; jgi| Gloin1| 78516|; jgi| Gloin1| 79920|
**Secondary metabolite-related**			
Up	3.84	29	jgi| Gloin1| 43670|; jgi| Gloin1| 322668|; jgi| Gloin1| 54382|; jgi| Gloin1| 334957|; jgi| Gloin1| 36234|; jgi| Gloin1| 22144|; jgi| Gloin1| 82149|; jgi| Gloin1| 17041|; jgi| Gloin1| 35874|; jgi| Gloin1| 74321|; jgi| Gloin1| 338850|; jgi| Gloin1| 20810|; jgi| Gloin1| 59731|; jgi| Gloin1| 57850|; jgi| Gloin1| 54172|; jgi| Gloin1| 60375|; jgi| Gloin1| 348593|; jgi| Gloin1| 330761|; jgi| Gloin1| 70184|; jgi| Gloin1| 343651|; jgi| Gloin1| 347608|; jgi| Gloin1| 54229|; jgi| Gloin1| 337700|; jgi| Gloin1| 348383|; jgi| Gloin1| 1959|; jgi| Gloin1| 83047|; jgi| Gloin1| 67532|; jgi| Gloin1| 2086|; jgi| Gloin1| 5484|
Down	–3.43	9	jgi| Gloin1| 130232|; jgi| Gloin1| 17438|; jgi| Gloin1| 21462|; jgi| Gloin1| 22126|; jgi| Gloin1| 300912|; jgi| Gloin1| 349273|; jgi| Gloin1| 51235|; jgi| Gloin1| 62820|; jgi| Gloin1| 71955|
**BTP/POZ and Kelch domain-containing**			
Up	10.70	8	jgi| Gloin1| 19708|; jgi| Gloin1| 15897|; jgi| Gloin1| 28179|; jgi| Gloin1| 335861|; jgi| Gloin1| 20150|; jgi| Gloin1| 6286|; jgi| Gloin1| 347430|; jgi| Gloin1| 33301|
Down	–4.26	3	jgi| Gloin1| 22970|; jgi| Gloin1| 336766|; jgi| Gloin1| 337622|
**Transporters and ion channels**			
Up	3.71	17	jgi| Gloin1| 12610|; jgi| Gloin1| 13617|; jgi| Gloin1| 22239|; jgi| Gloin1| 163857|; jgi| Gloin1| 23158|; jgi| Gloin1| 347430|; jgi| Gloin1| 75317|; jgi| Gloin1| 234547|; jgi| Gloin1| 345912|; jgi| Gloin1| 22144|; jgi| Gloin1| 17041|; jgi| Gloin1| 49664|; jgi| Gloin1| 338850|; jgi| Gloin1| 17710|; jgi| Gloin1| 336277|; jgi| Gloin1| 338361|; jgi| Gloin1| 144344|; jgi| Gloin1| 289764|
Down	–4.10	5	jgi| Gloin1| 15318|; jgi| Gloin1| 2573|; jgi| Gloin1| 343898|; jgi| Gloin1| 193343|; jgi| Gloin1| 298419|; jgi| Gloin1| 346110|

### Specific Responses to Nematode Infection in Galls From Mycorrhizal and Non-mycorrhizal Roots

During the compatible interaction, RKN trigger complex morphological and physiological changes in parenchymatic cells of the vascular cylinder to establish multinucleate feedings cells, called giant cells (GC), which serve as nutrient sinks for feeding. Nematode feeding sites, surrounded by cortical and epidermal cells, appear in the host root as the typical root-knot or gall.

**FIGURE 3:**
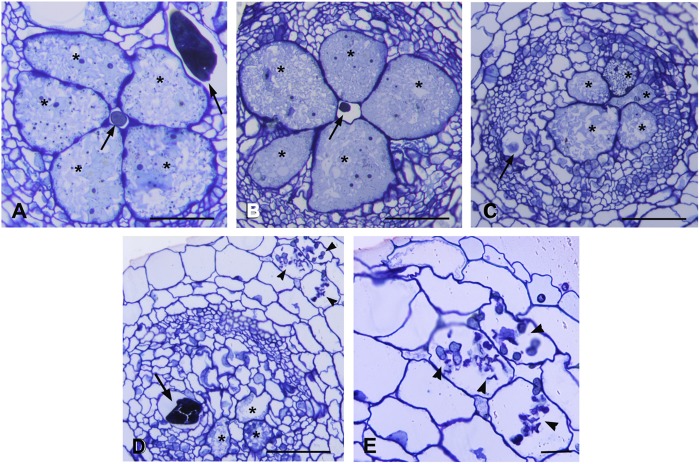
Histological analysis of feeding sites induced by *Meloidogyne incognita* in non-colonized and in *Rhizophagus intraradices*-colonized tomato roots at 7 days post inoculation. Cross sections (2.5 μm) were stained with toluidine blue and observed at light microscope. **(A)** Non-colonized gall contained well developed and metabolically active giant cells surrounding the nematode. **(B)** Giant cells in a AM-colonized gall with an appearance similar to those in non-colonized gall. **(C,D)** Feeding sites in AM-colonized roots presented giant cells with degraded cytoplasm and clear symptoms of early senescence. Note the presence of numerous hyphae in cortical cells **(D,E)**. Asterisk, giant cell; arrow, nematode; arrowhead, hyphae. Scale bars: 100 μm in **(A–D)** and 50 μm in **(E)**.

Microscopic observations were carried out on serial cross sections of AM-colonized and non-colonized galls at 7 dpi. Morphological changes were analyzed to monitor possible alterations in the development of the nematode feeding sites. Non-colonized galls showed large multinucleate GCs occupying the most part of vascular cylinder. The GCs presented small vacuoles and a dense granular cytoplasm containing numerous organelles, acting as a food sink for the growing nematode (Figure 3A). Several sections of AM-colonized galls revealed different features of the feeding sites (Figures 3B–D). Some of them showed GCs comparable with those of non-colonized galls (Figure 3B). Where AM fungal hyphae were observed near to the nematode feeding site (Figures 3D,E), GCs showed scarce cytoplasm with fewer organelles and clear symptoms of early senescence (Figures 3C,D). AM presence supported expression data of AM symbiosis marker genes and reads belonging to the fungus itself in this infection structure. In order to understand the tomato response during AM_RKN interaction, namely the impact of the symbiosis on the parasitism, data analysis was focused on a subset of genes already reported as specifically involved during susceptible RKN-tomato interaction (Iberkleid et al., 2015; Shukla et al., 2018).

AM-symbiosis differentially modulated the expression level of a set of genes involved in the parasitism response (Supplementary Table S12). DEGs (*P* > 0.05) showing an increased transcription level included those related to cell wall (8%), root development (25%), transport (33%), secondary metabolism (13%), hormone (15%), oxidative stress (21%), and stress response (4%). Down-regulated transcripts were mostly related to cell wall (23%), secondary metabolism (13%), hormone (17%), oxidative stress (10%), and stress response (4%) (Supplementary Table S12). The effect on the plant gene expression was evaluated calculating the ratio between AM_RKN/RKN and RKN/AM_RKN fold changes. Genes with ratio > |1.5| (for both up- or down- regulated) were considered in subsequent analyses (Table 9). The expression of all members from categories “root development” (Solyc04g078470.2.1, Solyc01g107710.2.1, Solyc05g009320.2.1) and “transport” (Solyc02g071070.2.1, Solyc04g079510.1.1, and Solyc09g074230.2.1) was induced by the fungus in galls (Supplementary Table S12), and among them the root cap protein 3 (Solyc01g107710.2.1) showed the highest ratio (11.95). Members of other categories (cell wall, hormone, secondary metabolite, oxidative stress response, and stress response-related genes) showed instead an uneven behavior (Table 9). The fungus colonization reduced the expression (negative ratio) of several genes involved in cell-wall metabolism, i.e., those acting on pectin fraction (Solyc03g083730.1.1 and Solyc04g076660.2.1, Solyc09g098270.2.1), two genes coding for an endo-1,4-b-xylanase (Solyc04g077190.2.1) and an endo-1,4-mannosidase (Solyc10g074920.1.1). By contrast, it positively affected the expression of three genes for putative expansins (Solyc07g054170.2.1, Solyc01g090810.2.1, Solyc05g007830.2.1). Among hormone category, genes coding for ethylene-responsive transcription factors (Solyc07g042230.1.1, Solyc08g007820.1.1, Solyc08g007830.1.1, Solyc11g042560.1.1 and Solyc10g050970.1.1) were negatively affected, except Solyc04g012050.2.1. Interestingly, the *CCD7* gene (Solyc01g090660.2.1), which is considered also an AM-symbiosis marker, was induced also in colonized galls. Among the genes involved in stress response some members of the cortical cell-delineating protein family (Solyc08g079230.1.1, Solyc08g078900.1.1 and Solyc12g014620.1.1) (Supplementary Table S12) appeared down-regulated in AM-colonized galls whereas others (Solyc03g091020.1.1, Solyc08g079200.1.1) were positively affected by the symbiosis.

**TABLE 9:** Effect of AM colonization on gene expression in galls, compared to untreated control (fold changes).

	**Transcript ID**	**Annotation**	**RKN**	**RKN_AM**	**RKN_AM/RKN**	**RKN/RKN_AM**
Cell wall	Solyc01g090810.2.1	Expansin protein	42.91	203.61	**4.74**	
	Solyc05g007830.2.1	Expansin 2	16.32	33.62	**2.06**	
	Solyc07g054170.2.1	Expansin B1	14.97	31.29	**2.09**	
	Solyc03g083730.1.1	Pectinesterase	–4.10	–6.95	**−1.70**	
	Solyc04g076660.2.1	Rhamnogalacturonate lyase	4.37	2.69		**1.63**
	Solyc04g077190.2.1	Endo-1 4-beta-xylanase	12.86	5.56		**2.31**
	Solyc09g098270.2.1	Polygalacturonase	31.62	19.02		**1.66**
	Solyc08g007090.1.1	Expansin-like protein	8.37	3.56		**2.35**
	Solyc10g074920.1.1	Mannan endo-1 4-beta-mannosidase	13.07	5.67		**2.31**
Root Development	Solyc04g078470.2.1	Cyclin D3-1	6.26	12.73	**2.03**	
	Solyc01g107710.2.1	Root cap protein 3	–76.43	–6.40		**−11.95**
	Solyc05g009320.2.1	LOB domain protein 42	–14.19	–6.14		**−2.31**
Transport	Solyc02g071070.2.1	Cation/H	–8.65	–4.15		**−2.08**
	Solyc04g079510.1.1	Peptide transporter	–27.47	–9.44		**−2.91**
	Solyc09g074230.2.1	Glucose transporter 8	–7.25	–2.69		**−2.69**
	Solyc11g008200.1.1	Nodulin-like protein	–5.79	–3.71		**−1.56**
Secondary metabolism	Solyc12g042460.1.1	4-coumarate CoA ligase	–18.22	–10.96		**−1.66**
	Solyc10g076660.1.1	Anthocyanidin synthase	–14.15	–8.39		**−1.69**
	Solyc10g076670.1.1	Anthocyanidin synthase	–12.11	–7.95		**−1.52**
	Solyc10g076680.1.1	1-aminocyclopropane-1-carboxylate oxidase	–10.54	–5.24		**−2.01**
	Solyc06g073580.2.1	1-aminocyclopropane-1-carboxylate oxidase 1	80.32	46.66		**1.72**
	Solyc01g009370.1.1	Cytochrome P450	27.77	17.79		**1.56**
	Solyc05g051010.2.1	Dihydroflavonol 4-reductase family	98.83	54.11		**1.83**
	Solyc05g051020.2.1	Dihydroflavonol 4-reductase family	24.33	14.51		**1.68**
Hormone	Solyc03g096050.2.1	1-aminocyclopropane-1-carboxylate oxidase 1	–8.49	–5.19		**−1.63**
	Solyc04g012050.2.1	Ethylene responsive transcription factor 2a	–8.97	–5.94		**−1.51**
	Solyc05g006220.2.1	IAA-amino acid hydrolase	–10.87	–5.07		**−2.14**
	Solyc02g077430.2.1	Lipase-like	–3.95	–2.53		**−1.56**
	Solyc08g006860.2.1	Patatin	–8.97	–5.47		**−1.64**
	Solyc09g075870.1.1	Lipoxygenase	–4.47	–2.94		**−1.52**
	Solyc01g090660.2.1	Carotenoid cleavage dioxygenase 7	–18.87	–5.30		**−3.56**
	Solyc07g042230.1.1	Ethylene-responsive transcription factor 7	–3.73	–6.95	**−1.86**	
	Solyc07g049530.2.1	1-aminocyclopropane-1-carboxylate oxidase	–3.03	–5.52	**−1.82**	
	Solyc08g007820.1.1	Ethylene-responsive transcription factor 10	–6.07	–11.81	**−1.95**	
	Solyc08g007830.1.1	Ethylene-responsive transcription factor 10	–14.92	–24.04	**−1.61**	
	Solyc10g050970.1.1	Ethylene responsive transcription factor 2b	–9.49	–17.23	**−1.82**	
	Solyc11g042560.1.1	Ethylene-responsive transcription factor 4	17.78	10.49		**1.70**
	Solyc01g109140.2.1	Cytochrome P450	–2.24	–4.17	**−1.86**	
	Solyc04g007790.2.1	Major latex-like protein	154.36	81.88		**1.89**
Oxidative stress	Solyc02g064970.2.1	Peroxidase	–9.34	–3.51		**−2.66**
	Solyc03g116120.1.1	Glutathione *S*-transferase 12	–24.40	–8.02		**−3.04**
	Solyc05g006740.2.1	Glutathione *S*-transferase	–17.48	–3.41		**−5.13**
	Solyc05g006750.2.1	Glutathione *S*-transferase 1	–7.57	–3.30		**−2.30**
	Solyc05g006860.2.1	Thioredoxin H	–17.81	–11.33		**−1.57**
	Solyc05g046000.2.1	Peroxidase 27	–19.32	–3.59		**−5.37**
	Solyc02g083480.2.1	Peroxidase	10.13	3.99		**2.54**
	Solyc12g094620.1.1	Catalase	9.52	5.82		**1.63**
	Solyc06g082420.2.1	Peroxidase 3	–15.99	–40.81	**−2.55**	
Stress response	Solyc03g091010.1.1	Cortical cell-delineating protein	–47.01	–10.25		**−4.59**
	Solyc03g091020.1.1	Cortical cell-delineating protein	–11.41	–5.92		**−1.93**
	Solyc08g079200.1.1	Cortical cell-delineating protein	–5.94	–3.31		**−1.79**
	Solyc08g079230.1.1	Cortical cell-delineating protein	25.45	13.23		**1.92**
	Solyc08g078900.1.1	Cortical cell-delineating protein	6.52	4.23		**1.54**
	Solyc12g014620.1.1	Cortical cell-delineating protein	333.57	178.73		**1.87**
	Solyc06g053950.1.1	Heat stress transcription factor A-2c	–4.14	–7.43	**−1.80**	
	Solyc10g086680.1.1	Class I heat shock protein. HSP20-like chaperone	–4.63	–8.45	**−1.83**	

*Ratios were calculated by using RKN_AM and RKN data. Only ratios > |1.5| are reported.*

## Discussion

Several studies addressed the effect of water stress on growth, yield, secondary metabolite production and gene expression in tomato (Nuruddin et al., 2003; Sánchez-Rodríguez et al., 2011; Giannakoula and Ilias, 2013; Sacco et al., 2013; Iovieno et al., 2018). Previous works also studied some specific aspects of the relationship between tomato and AM fungi under drought stress (Dell’Amico et al., 2002; Subramanian et al., 2006; Aroca et al., 2008; Wang et al., 2014; Chitarra et al., 2016; Ruiz-Lozano et al., 2016; Volpe et al., 2018). An untargeted metabolomic analysis of tomato roots colonized by three AM fungi of different genera, verifying their impact on tolerance to drought or salt stress during symbiosis, showed DEGs from several processes, by looking at genome-wide transcriptional changes (Rivero et al., 2018). Massive transcriptional changes are known to occur during AM symbiosis both in dicots and monocots, and a core of marker genes, considered the functional signature of AM symbiosis, have been identified (Guether et al., 2009; Sugimura and Saito, 2017; Fiorilli et al., 2018).

Previous experiments, using the same biological system and water stress level herein studied, showed that *R. intraradices* colonization did not lead to a significant difference in plant performance traits. Tomato plants inoculated with *R. intraradices* showed enhanced internodes/height ratios under water deficit, suggesting a positive AM influence in water stress regimes, likely due to a more compact plant architecture less subject to water dispersion (Volpe et al., 2018). However, in agroecosystems, in addition to water limitation, crops often face other concurrent abiotic and biotic stresses, e.g., insect pests and pathogens (Bai et al., 2018). In the same study, *R. intraradices* appeared effective in sustaining the plant response to a combined abiotic (moderate WS) and biotic stress (aphid attack), the latter in terms of attractiveness toward aphid natural enemies (Volpe et al., 2018). The impact of AM symbiosis on the transcriptomic profile of nematode infection structure (i.e., the gall), firstly confirms the regulation in AM-colonized plants of diverse metabolic pathways, such as primary and secondary metabolisms, ion transport, transcriptional regulation, including several AM symbiosis markers.

Our dataset emphasizes the putative role of specific gene families during symbiosis establishment and functioning. For example, five out of the six genes coding for putative ripening-related proteins (RRP) were strongly significantly up-regulated both in AM-colonized unstressed and water stressed plants (with FC from 4000 to 395 in AM and from 1000 to 38 in AM_WS). Although their function during symbiosis have not been demonstrated, RRP are known to be regulated in several plant/AM combinations (Fiorilli et al., 2015, 2018; Handa et al., 2015). Genes belonging to this family have been found to be strongly induced in rice large lateral roots colonized by the AM fungus (Fiorilli et al., 2015), including *OsAM8*, previously identified as a mycorrhiza-responsive gene (Güimil et al., 2005). Additionally, RRP have been reported among the cysteine-rich peptides highly induced genes in AM *Lotus* roots (Handa et al., 2015). The expression of several *Medicago* RRP was activated, with respect to wild type plants, in a mutant (*pt4*) that leads to early arbuscule degeneration (Floss et al., 2017), opening new questions on the role during symbiosis.

Other gene families showed almost all members as significantly regulated in AM-colonized roots (e.g. blue copper-binding proteins, which are considered markers for AM symbiosis and include phytocyanins that play an important role in plant development and stress resistance), suggesting a functional redundancy as already demonstrated for phosphate transport. However, the location of their regulation remains still unknown (i.e., arbuscule-containing cells *vs.* non-colonized ones). Looking at nutritional aspects, it is worth noting the huge number of up-regulated transporter genes, that indirectly confirm the symbiosis functionality. Interestingly, most of them were significantly up-regulated in AM_WS roots, suggesting that the symbiosis was still functional after imposition of the water stress. The lower gene expression levels in comparison to those in AM roots is in agreement with a decrease in the colonization rate previously reported during water deficit (Chitarra et al., 2016) and also confirmed in this work by a lower percentage of AM fungal reads in AM_WS roots with respect to unstressed plants (AM). However, although symbiosis seems to be affected by water limitation, the impact on root transcriptome is evident also in AM_WS roots. This result reflects the fact that a functional symbiosis seems to be still present during the progression of the water stress, at least for the plant/fungus combination considered in this study.

The 20 most up-regulated genes in AM_WS, compared with those involved in regulation of stress, indicate that AM-colonized roots may differently “sense” the water stress with respect to the uncolonized ones. The most up-regulated genes were in fact still those involved in the symbiosis, at least upon a moderate stress.

Regulation of stress marker genes (i.e., LEA, dehydrin, ABA-responsive genes, P5CS), as observed in AM_WS roots, confirmed the effect of the imposed stress.

The physiological plant response to water deficit is also regulated by the aquaporin proteins (AQPs), controlling water movement through the plant in different physiological conditions. Their regulation is considered as an adaptative mechanism to stress conditions (Kapilan et al., 2018). Molecular analyses already demonstrated a complex transcriptional and post-transcriptional regulation. Data from several studies indicate that AM symbiosis has an impact on host AQPs, altering both plant-water relationships and plant physiology, to better cope with stressful conditions such as drought. However, as reported for other functional aspects related to AM symbiosis, the regulation of AQPs seems to be dependent on the plant and fungal species involved in the symbiosis (Ruiz-Lozano and Aroca, 2017). Data from AM-colonized maize roots, exposed to several growing and water-stressed conditions, showed that the fungus may regulate a large number of AQP genes in the host plants, in several sub-families. Regulation, however, was also dependent on water status and the duration and severity of the imposed stress (Ruiz-Lozano and Aroca, 2017; Balestrini et al., 2018). This can explain the different result with respect to the data already reported by Chitarra et al. (2016) in the same plant/AM fungus combination, but upon a severe water stress level.

If relevant data are nowadays available on the plant protective effect of AM fungi under water stress, the impact of drought on the fungus itself has not been extensively investigated. Our dataset revealed that cytochrome P450 (CYPs) genes were mainly up-regulated in *R. intraradices*, in presence of water stress. Fungi possess many diverse CYPs, mainly involved in sterol biosynthesis, developmental processes and production of secondary metabolites such as those involved in virulence (Shin et al., 2018). The *R. irregularis* genome, used for the mapping of fungal genes, contains about 200 CYP-encoding genes, indicating an expansion of such a gene family in AMFs that likely deals with the peculiar AM lifestyle (Tisserant et al., 2013; Handa et al., 2015). As cytochrome P450 is involved in the synthesis of sterols for membrane biogenesis during arbuscule formation (Handa et al., 2015), it could be inferred that their over expression reflects a change in fungal development under water deficit. Other most highly up-regulated fungal genes include a “conidiation protein 6” domain (*con-6*). In *Neurospora crassa con-6* encodes a small protein expressed during the formation of conidia (White and Yanofsky, 1993). Fungal conidiation can be induced by nutrient deprivation or mycelium desiccation, a process worth to verify for *R. irregularis* propagules upon drought. The sequences most DE in our database included several that were poorly characterized. This situation may reflect the lack of a functional annotation for a consistent part of the AMF genomes so far sequenced, but might also underlie unprecedented, unknown mechanisms exploited to cope with water stress. Several sequences encoding for stress response-related proteins were regulated, suggesting an impact of the water deficit on the AM fungal metabolism, but their mechanism of action could not be hypothesized, based on current annotation data. Fungal genes containing domains involved in signaling transduction, regulation at the protein level and protein turnover were significantly influenced by the WS treatment, being mainly up-regulated. Such gene families underwent a significant expansion in the *R. irregularis* genome, likely due to the biotrophic lifestyle of the fungus (Zuccaro et al., 2014). Our current data strengthen this idea, suggesting that BTB/POZ and Kelch domain-containing proteins might play a central role in the fungal sensing of environmental stimuli, including the perception of and the response to abiotic stresses.

Three glutathione *S*-transferases (GSTs) were overexpressed in *R. intraradices* upon water stress. GSTs are acknowledged players in the cell protection from oxidative damage, and in tomato one GST is involved in osmotic and salt stress tolerance (Xu et al., 2015). Taken together, these data suggest that the up regulation of GST-encoding genes might represent a conserved hallmark of water stress response, from plants to fungi. Additionally, this confirms previously results on the fact that specific categories of genes involved in stress response can be activated in both the symbiotic partners, such as for AQP (Chitarra et al., 2016) and mitogen-activated protein kinase (MAPK) genes (Liu Z. et al., 2015), in tomato and soybean, respectively.

Plant responses to different stresses are highly complex and dynamic and involve changes at the transcriptome, cellular, and physiological level that are related to the specific environmental stress factors encountered. Only a few data are still available on the molecular basis of AM symbiosis-induced resistance against nematodes (Hao et al., 2012; Vos et al., 2012). Data from our study allowed the identification of a core of tomato DEGs related to parasitism response, essential for a successful RKN-tomato association, that was differently modulated in galls from mycorrhizal colonized roots.

RKN induced GCs, that are characterized by cytoskeleton rearrangements, a fragmented vacuolar system, and an increased cytoplasm density with many organelles, may be 100 times as large as normal root parenchyma cells. Thus, an extensive, coordinated remodeling of the cell wall must occur to allow cell expansion (Williamson and Hussey, 1996; Gheysen and Mitchum, 2011). Our data showed different responses in genes associated to cell wall modification in AM-colonized galls.

Several transcripts coding for proteins involved in cell wall biosynthesis and modification were significantly down-regulated in AM-colonized *vs.* non-colonized galls, suggesting that AM colonization might induce changes in nematode feeding sites by counteracting cell expansion. Conversely, three expansin genes up-regulated during susceptible interaction were highly over expressed in colonized galls. Plant expansins are cell wall loosening proteins involved in the extension of the cell wall (Sampedro and Cosgrove, 2005) and may have a role in the establishment of RKN in tomato (Gal et al., 2006). They have been also reported to be involved in the AM fungal symbiosis development, at different stages of the interaction (Balestrini et al., 2005; Dermatsev et al., 2010). Therefore, we can hypothesize that the highest expression of these genes in *R. intraradices* colonized galls could be explained by the combined action of RKN infection and AM fungal colonization.

The presence of the AM fungus affected other pathways involved in oxygen species (ROS) homeostasis, and cell cycle/root development and response to stress (Table 7), which can have a role during the establishment of compatible plant-nematode interaction. Nematodes modulate the production of plant ROS that otherwise would be detrimental for their development. Plant peroxidases (POXs) have an important role in ROS homeostasis as they oxidize phenolics, lignin precursors, auxins and secondary metabolites using hydrogen peroxide (Almagro et al., 2009). In addition to the generation/scavenging of ROS, peroxidase activities have been also associated to loosening/stiffening of the cell wall (Shigeto and Tsutsumi, 2016). Due to the large set of peroxidase isoforms, it is difficult to determine their role in the different biological processes and especially in plant defense response. In the compatible tomato-*M. incognita* interaction genes coding for peroxidases are up- or down-regulated at the same infection time, suggesting that each individual enzyme has its own unique physiological and developmental role (Melillo et al., 2014). Likewise, in galls from AM-colonized roots some genes coding for POXs were differently regulated with respect to galls from non-colonized plants. Peroxidase genes were also regulated in AM-colonized soybean roots, in presence of infection by *Fusarium virguliforme* (Marquez et al., 2018). The decreased down-regulation of several genes coding for glutathione-*S*-transferase (GST) in galls from AM-colonized plants compared to non-colonized ones suggests a putative protective role of GST against nematode damage, as previously reported during the *R. intraradices*-induced biocontrol of the ectoparasitic nematode *Xiphinema index* in grapevine (Hao et al., 2012).

Members of heat shock protein families (Hsp100, Hsp90, Hsp40, and Hsp20) were down-regulated in RKN and RKN-AM galls, suggesting that plant machinery related to Hsp families is silenced and other pathways may be activated. Interestingly, members of the plant non-specific lipid transfer proteins (LTP), classified as pathogenesis-related proteins, were modulated in the same way in RKN and RKN-AM galls, suggesting different functions in several physiological and stress pathways (Edqvist et al., 2018). Recently, it has been reported that some LTP can induce secondary messengers involved in the induction of different signaling proteins (MAPK family, heat shock factors, etc.) (Li et al., 2014). Another class of LPT is that of the cortical cell delineating proteins, which accumulate in normal root tips and are responsible in the responses of the roots to physical impedance (Huang et al., 1998). Our results showed that they were differentially regulated in RKN and RKN_AM galls and, in particular, the significant increase of the expression of three genes in RKN_AM galls could suggest a protective role of AM in root defense. Other genes associated with stress responses are those encoding major latex protein family (MLP), active in a wide range of developmental processes and in response to biotic and abiotic stresses (Sun et al., 2010; Yang et al., 2015). In our study the Solyc04g007790.2.1 was strongly up-regulated in RKN galls, while in RKN_AM galls the level of this MLP was reduced but still up-regulated, suggesting a role of this protein in the defense response to *M. incognita*, and that AM could activate other defense pathways.

Galls also showed a different modulation of genes involved in root development and cell cycle, in presence of AM. Expression of cell cycle genes in the plant host appears tightly regulated, implying a strict control of the cell cycle machinery and its molecular components during the plant–nematode interaction (Vieira and de Almeida Engler, 2015, 2017). Our results suggested that in galls from AM-colonized roots a different modulation of gene related to the cell cycle, root tip and lateral root development takes place, indicating a transcriptional reprogramming of the root development, also affected by the fungus. By antagonizing the host plant immune response, RKN manipulate defense pathways in the root galls to promote their own development. Our data agree with previous studies stating that genes involved in JA and ET biosynthesis and signaling are suppressed during fully established RKN compatible interaction (Barcala et al., 2010; Nahar et al., 2011). Several genes involved in JA and ET biosynthesis were in fact less down-regulated by AM, suggesting a complex interaction mediated by the fungus.

Additionally, the carotenoid-derived phytohormones strigolactones (SLs) can enhance symbiosis between plants and AM fungi by inducing hyphal branching (Akiyama et al., 2005) and are also important in tomato defense against RKNs (Xu et al., 2019). It was in fact observed that the silencing of *CCD7*, coding for an enzyme involved in SLs biosynthesis, increased plant susceptibility to RKNs (Xu et al., 2019). Our data showed that *CCD7* was strongly down-regulated in non-colonized galls, while it appeared to be induced in galls from AM-colonized roots, in agreement with their known role in AM-symbiosis, associated to early senescence of AM-colonized galls. In presence of the RKN, a suppression of plant defense responses during parasitism has been also observed, involving an alteration of secondary metabolism. Conversely, in galls from AM-colonized roots, transcription of 4-coumarate CoA ligase and anthocyanidin synthase, i.e., two enzymes involved in the flavonoid pathway leading to anthocyanins and condensed tannins, was activated. Flavonoids act during plant-nematode interactions as defense compounds or signals affecting nematode fitness at different life stages (Chin et al., 2018). They may also be involved in nematode feeding site development by maintaining local auxin accumulation. Therefore, the induction of these two enzymes as well as the reduction of the expression of genes belonging to dihydroflavonol-4-reductase family, up-regulated also in other plant–pathogen interactions (Liu et al., 2010), suggest that AM colonization help plants to respond to nematode challenges.

## Conclusion

Our transcriptome dataset offers new information about the symbiotic-responsive genes both from tomato and the AM fungus, representing a solid basis for future investigations. Moreover, data provided new information on the water stress perception by AM-colonized roots as well as on the response to a biotic stress. Several marker genes (for both AM-colonization and stress factors) have been identified, confirming the robustness of the obtained dataset. Lastly, results on the regulation of AM fungal genes upon a moderate water stress condition have been obtained, suggesting a synergy between plant and fungus in this condition. Sequencing data and morphological observations also indicate that the mechanisms involved in the tomato responses to nematode colonization may also be mediated by the AM symbiosis.

## Author Contributions

RB, LR, AC, and IP conceived and designed the experiments, and oversaw the manuscript preparation. LR, PV, and MC performed the RNA extraction. PV and MM performed the histopathology and morphological observations. IP performed the bioinformatics analyses. RB, LR, PV, EF, FDL, AdF, and IP performed the data analysis. RB, LR, PV, and AC wrote the manuscript. All authors read and approved the final manuscript.

## Conflict of Interest Statement

The authors declare that the research was conducted in the absence of any commercial or financial relationships that could be construed as a potential conflict of interest.

## Footnotes

1 www.igatechnology.com

2 https://www.encodeproject.org/about/experiment-guidelines/

3 https://www.bioinformatics.babraham.ac.uk/projects/fastqc

4 http://www.clcbio.com

5 ftp://ftp.solgenomics.net

6 http://bioinfo.cau.edu.cn/agriGO

7 ftp://ftp.solgenomics.net/tomato_genome/annotation/ITAG2.4_release/ITAG2.4_cdna.fasta

8 https://genome.jgi.doe.gov

## Supplementary Material

FIGURE S1 Specific GO terms enriched during abiotic stress (AM_WS).

FIGURE S2 Specific GO terms enriched during biotic stress (RKN_AM).
