# Supplementary figures and images for: Transcriptomic Responses to Water Deficit and Nematode Infection in Mycorrhizal Tomato Roots
Source: Front Microbiol. 2019 Aug 13;10:1807. doi: 10.3389/fmicb.2019.01807 (PMC6700261; doi:10.3389/fmicb.2019.01807)

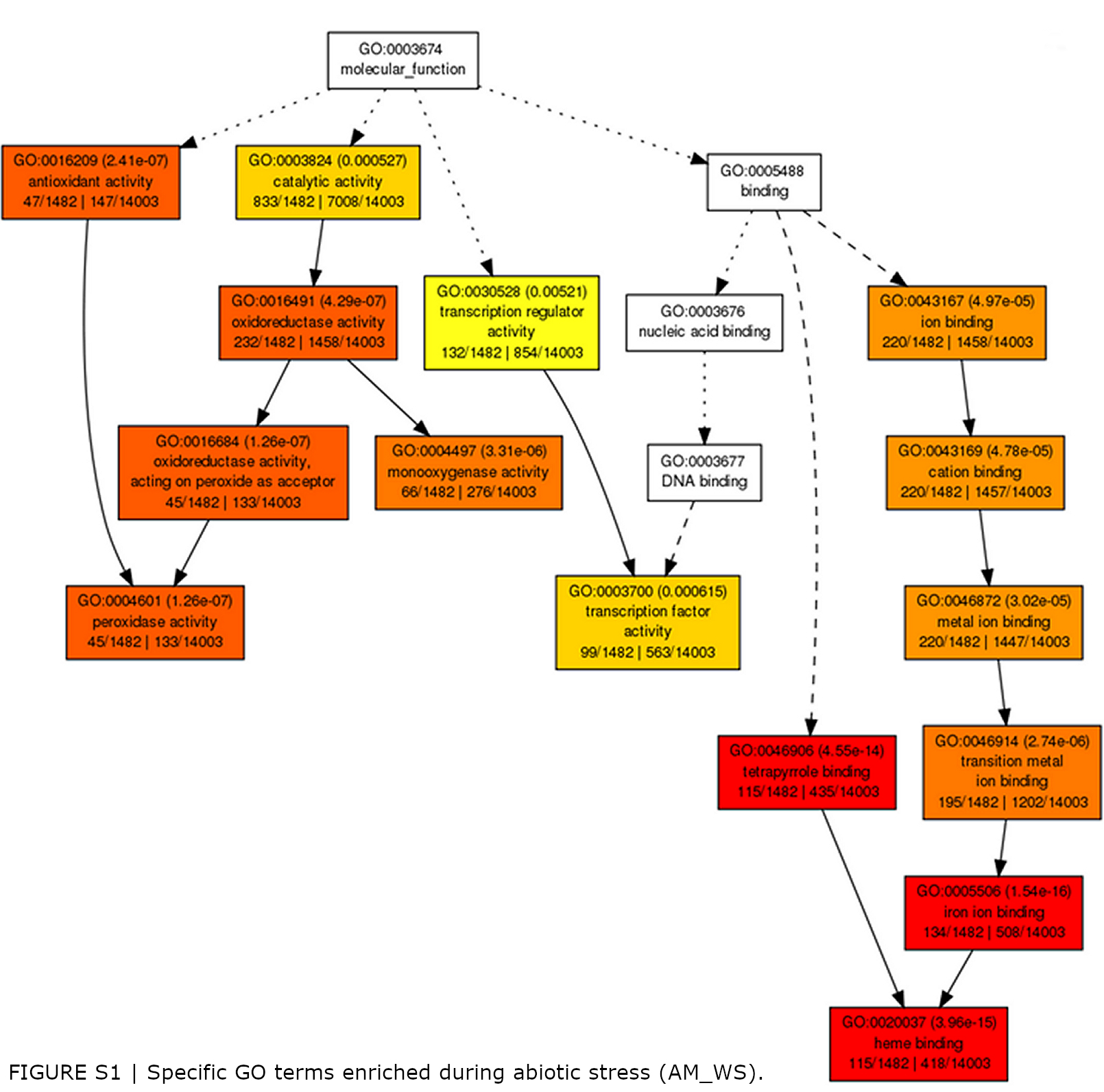

Supplement: FIGURE S1 — Specific GO terms enriched during abiotic stress (AM_WS). [file Image_1.jpg]
